# Validity and feasibility of a predictive language screening tool in 2-year-old children in primary pediatric care

**DOI:** 10.3389/fped.2022.865457

**Published:** 2022-09-06

**Authors:** Daniel Holzinger, Christoph Weber, Johannes Fellinger

**Affiliations:** ^1^Institute of Neurology of Senses and Language, Hospital of St. John of God, Linz, Austria; ^2^Research Institute for Developmental Medicine, Johannes Kepler University Linz, Linz, Austria; ^3^Institute of Linguistics, University of Graz, Graz, Austria; ^4^Department for Inclusive Education, University of Education Upper Austria, Linz, Austria; ^5^Division of Social Psychiatry, University Clinic for Psychiatry and Psychotherapy, Medical University of Vienna, Vienna, Austria

**Keywords:** language screening, predictive, two-year olds, late language emergence, late talker

## Abstract

**Objective:**

To assess the predictive validity and feasibility of the newly developed language screening tool, SPES-2 (Sprachentwicklungsscreening), for 2-year-old children in pediatric primary care.

**Methods:**

A prospective cohort study recruited 2,044 non-selected German-speaking children undergoing a regular well-baby check-up at the age of 2 years. Thirty primary care pediatricians spread over urban and rural areas screened the children using a short parent-reported questionnaire and direct assessment of word comprehension. To validate the screening tool, language skills were assessed using a standardized language screening tool in the complete sample 1 year later. Data of a random sample of 621 children were analyzed. Feasibility of the screening tool was evaluated using questionnaires completed by the participating pediatricians.

**Results:**

The new screening tool, SPES-2, demonstrated good diagnostic accuracy with AUC (Area under the Roc Curve) of 0.885, a sensitivity of 0.74, and specificity of 0.86, using a parent-reported questionnaire (expressive vocabulary, two-word combinations, parental concerns) as stage 1, followed by a stage 2 direct assessment of word comprehension by the pediatrician. The second stage was restricted to children who failed the parental screening. The screening identified children with high, moderate, and low risk of significant language deficits (SLD) at the age of 3 years, permitting tailored follow-up assessment and parental counseling. Practicality and acceptability of the screening were mostly rated as high. Pediatricians regarded the availability of follow-up diagnostic services and parent guidance as most important for a general implementation of the new instrument.

**Conclusion:**

The language screening tool, SPES-2, was valid for the identification of significant language deficits 1 year later, and considered as feasible within primary pediatric care.

## Introduction

Language disorders are among the most frequently diagnosed developmental disorders among young children, with reported prevalence rates of about 10%. These disorders may be of unknown origin (7.58%) or associated with other developmental disorders, such as general cognitive delay or autism spectrum disorder (1, 2). Children with language difficulties are at increased risk of adverse outcomes, including social isolation, mental health problems (3–5), academic problems (6, 7), placement in special education, and later unemployment (8, 9). Therefore, language problems affect the functioning of individual children and represent a loss for society.

Increasing evidence shows that early intervention—particularly if family-centered—is effective in improving language outcomes among those with language (10–15) difficulties (10–15).

As early identification and subsequent intervention may significantly influence functional outcomes in children, systematic developmental surveillance has been recommended by the American Academy of Pediatrics (16, 17). However, in a systematic review, Nelson et al. (18) indicated a lack of valid language screening instruments. Almost one decade later, Robins et al. (19) reported several screening tools that could accurately identify children with language disorder, but with insufficient evidence of feasibility in the pediatric primary care setting. As a result, the U.S. Preventive Task Force did not recommend population screening for language disorders (20). Following international reviews, the German Institute for Quality and Efficiency in Health Care (21) considered the available evidence insufficient for a recommendation of universal language screening in Germany.

The development of an accurate and feasible screening tool is complicated by the instability of developmental trajectories during the early years. By the age of 3–4 years, about half of the children with late language emergence at the age of two attain age-appropriate language levels (within one standard deviation) (22–24). In a national twin study in England and Wales (25), including 4,193 twin pairs, 56% of those with late language emergence did not meet criteria for persistent language disorder at the age of 3 years. Whereas some children show late language emergence, others manifest deterioration in the trajectory of language development over time. In Poll and Miller's (26) longitudinal study (*n* = 1,015), more than 60% of children with weak oral language skills at 8 years did not have a history of late language emergence at the age of 2 years. In the Early Language in Victoria Study with an epidemiological focus, less than half of the children with language disorder at the age of 4 years were identified as late talkers at the age of 2 years (27). The high rate of recovery from early language delay requires the development and validation of screening instruments predicting long term language outcomes (predictive validity). The emergence of later language disorder despite earlier typical performance suggests the necessity of continuous surveillance of language development.

The use of parent-reported or direct assessment is another critical issue in the development of an instrument screening for increased risk of language difficulties. In their systematic review on preschool screening tools for language and behavioral difficulties, Sim et al. concluded that parent-reported screening tools for language in preschool aged children achieved higher sensitivity, specificity, and negative predictive value than direct child assessment (28). However, Visser-Bochane et al. (29) reported high predictive validity for a screening instrument (van Wiechenschema) based on a combination of direct child assessment and parent report. Evaluations of parent-reported tools (MCDI and LDS) showed high specificity with moderate sensitivity, whereas the combined tool had poorer specificity but better sensitivity, and thus higher rates of prediction accuracy of children with language delays. In a study to compare the validity of parental screening and direct pediatric assessment of child development (30), direct professional assessment had a higher validity than parental reports. Therefore, parental reports may not necessarily be superior to direct observation or testing. The critical factor may be the availability of valid and easy-to-use instruments for language screening within the time constraints of primary pediatric care. Pediatricians have longitudinal relationships with children and families and therefore the child's entire medical history (31), facilitating interpretation and augmentation of screening based on parent report. By completing a short, direct observation of the child's language development, pediatricians may build trust that facilitates communication with families about their child's developmental delays.

A further essential characteristic of a modern screening instrument relates to the required resources for assessment and follow-up. 2-stage screeners collecting data from parents in the first step and requiring pediatric assessment in the second step exclusively with those failing the initial screening have been shown to be effective for the identification of autism (19). The majority of available language screening instruments do not include cut-offs that allow for stepped follow-up such as referral for diagnostic assessment or parent counseling for an exception see (29).

Language screening instruments that can be used within preventive medical care have been available for decades; examples of which include the MacArthur-Bates Communicative Development Inventories (MCDI) (32) and Language Development Survey (LDS) (33). In German-speaking countries, the ELFRA (Elternfragebogen) Parent Questionnaire (34), a parent-reported language screening tool with 260 items based on the MCDI and LDS, has been normed for 2-year-old children (35, 36). In addition, a shorter parent-reported word list (SBE 2 KT; Sprachbeurteilung durch Eltern Kurztest; a short language assessment test by parents) has been developed (37) and validated. The FRAKIS (Fragebogen zur kindlichen Sprachentwicklung; Questionnaire on child language development) (38) is another more extensive parent-reported questionnaire based on the MCDI that collects information on expressive vocabulary (600-item word list) and grammatical development. A short form of the FRAKIS with a word list of 102 items and three questions referring to grammatical development is available. A systematic review on the effectiveness of population-based language screening for children at pre-school age in Germany concluded that the accuracy of the screening instruments has not been sufficiently examined (39). Within the Austrian system of well-baby checkups, the screening protocol at the age of 2 years includes several questions on language development. However, neither cutoffs for non-typical development nor guidelines for subsequent referral are available. Therefore, no evaluations of the performance of the well-baby check-up for the identification of language delay in primary pediatric care in Austria have been published. Currently, none of the above mentioned instruments fulfills all of the following criteria: validation with a representative total population sample; validation within a community setting; follow-up including referral of children according to a strict referral protocol and subsequent systematic, standardized testing of a representative sample of children, independent of screening results; reporting predictive validity of the screening measures regarding language outcomes 1 year after the screening; and inclusion of parent report and pediatric assessment.

The Federal State of Upper Austria has a population of 1.45 million inhabitants and approximately 13,000–15,000 births annually within the last 20 years. Universal, free preventive medical care is available and provided primarily by pediatricians but also by general practitioners.

This study sought to establish and validate a language screening tool for 2-year-old children (i) in a representative comprehensive population sample; (ii) with high feasibility in routine primary care pediatric settings within the regular well-baby check-ups; (iii) predicting language problems 1 year after the screening; (iv) including a two-stage procedure of parent-reported assessment followed by pediatric direct assessment only in case of atypical results in the first screening stage, with the intention to include valuable parent information and to reduce the pediatricians' time required for direct assessment; (v) with sufficient sensitivity (identification of a high proportion of children with SLD at age three) and a high proportion of true positives of the screening positives (positive predictive value) to avoid costs for follow-up and unnecessary irritation of parents; and (vi) resulting in a graduation of risk levels of SLD (low, moderate, high) that allow for well-adjusted procedures following the screening.

## Methods and procedures

This study is part of a comprehensive pre-school language surveillance project with the aim to establish a language screening tool for children at the ages of 2 and 3 years in the whole State of Upper Austria. The project was implemented in close cooperation with the pediatric association of Upper Austria. An initial pilot study (2007–2008) aimed to identify screening components that predict language disorders about a year after the administration of the screening tool (predictive validity) and thus establish a screening tool. In the actual validation study (2009–2010), the predictive validity of the new screening tool was assessed and reported in this paper.

### Construction of the screening tool and pilot testing

The initial screening tool was constructed and implemented within a 2007–2008 pilot study (40) as a combination of a parent-reported questionnaire, child medical data available from the well-baby check-up, and direct pediatric assessment. A representative group of pediatricians (*n* = 30) across the State of Upper Austria participated in the study. The pediatricians were introduced to language development and language screening, and trained in the administration of the screening instruments. In the pilot study, the parent-reported questionnaire and direct pediatric assessment were administered to all children growing up with German as their primary family language. Children having another preferred family language than German and speaking German as their best language (and thus growing up multilingually) were also excluded from the pilot study.

The parent-reported questionnaire included (i) a short form of the ELFRA, [Elternfragebogen; Parent Questionnaire; (34)], a 260 items word list of the child's expressive vocabulary, (ii) a question on the child's use of two-word combinations, (iii) sociodemographic information of the family (parental education, the child's birth order), (iv) parental concerns about language development (typical, slightly delayed, severely delayed), (v) parental estimation of language development, (vi) family predisposition for language and literacy difficulties, and (vii) a history of otitis media. In addition, (viii) information about the primary language used in the family and the child's best language were collected. Medical data extracted from the regular documentation of the well-baby check-ups (Mutter-Kind-Pass; Mother-Child-Passport) included gestational age, multiple birth, APGAR (Appearance, Pulse, Grimace, Activity, and Respiration) scores, birth weight, size, head circumference, and preceding diagnoses. The pediatricians assessed word comprehension by asking the child to identify six body parts on a doll.

For children at the age of 3 years, the pediatricians performed two standardized subtests of a comprehensive German language test [SETK-3-5 (41)], assessing noun plurals and sentence comprehension. To determine predictive validity of the screening measures for children at the age of 2 years, a validation sample of 141 children (64 with ELFRA scores below 50, 29 with ELFRA scores between 50 and 79, and a group of 48 children with scores above 79) was assessed by speech-language experts blinded for the screening results by use of standardized tests, including noun plurals, non-word repetition, sentence comprehension, and encoding of semantic relations (SETK-3-5) and expressive vocabulary [AWST-R (42)]. Cases of children with significant language delay were extrapolated from the subsample of 141 children on the total sample of children with German as their preferred family language and complete data at the ages of 2 and 3 years (*n* = 1,543). This extrapolation was based on the correlation between the results of the language tests performed by speech-language experts and pediatricians.

In the pilot study, parent reports on expressive vocabulary (ELFRA 2) of children at the age of 2 years were found to be the best predictors of language status 1 year later. Other factors that added significant predictive quality were (in this sequence) sentence use of two-word utterances, parental concerns, parental estimation of language development, and the short pediatric assessment of word comprehension. Based on these findings, the screening procedure was reduced to a shortened parent-reported questionnaire and direct assessment of word comprehension by the pediatricians.

### Study procedures and recruitment

In 2009, the Pediatric Association of Upper Austria invited all primary care pediatric offices to participate in a longitudinal project on language screening. Thirty pediatricians (about 50% of all primary care pediatricians of Upper Austria) from across the country agreed to screen all children for language disorders at the ages of 2 and 3 years within their regular well-baby check-ups. The pediatricians and their office assistants were trained in the administration of the final screening procedures, scoring, and communication of results to parents, and provided with the screening materials.

**Figure 1:**
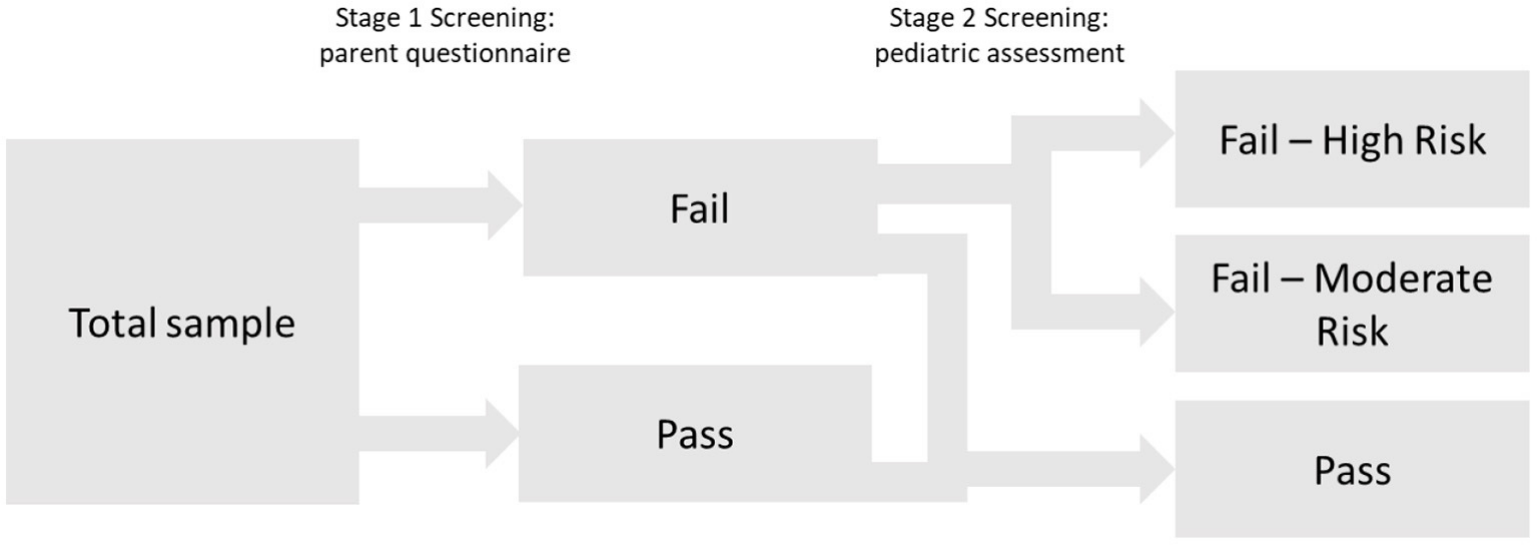
Conceptual overview of the two-stage screening tool.

In 2009, about 66% of the entire Upper Austrian birth cohort of 2007 (*n* = 13,297) were assessed during regular well-baby check-ups, 70% (6,200) of them by pediatricians and the others by general practitioners. This study used data from 2,044 children growing up with German as their primary family language screened at the ages 2 (23–25 months) and 3 years (35–7 months). This accounts for about 15% of the entire birth cohort and 33% of children examined by pediatricians. Overall, the sample was representative, regarding sex ratio and prematurity rate (see Table 1). Additionally, the distribution of maternal education was comparable to that of the population. Notably, the seeming overrepresentation of higher educated parents in the sample is likely due to the exclusion of non-German speaking children, whose parents have an lower education level than parents of German speaking children (45). Notably, the initially recorded data contained only the sum score of the 260-word expressive vocabulary list. To reduce the administration time by parents, a random sample (*n* = 667; see Figure 1) stratified by age, gender, and maternal education was drawn from the total sample; and for these children, all 260 items were separately entered in retrospect. The item reduction to a final list of 37 words was achieved by deleting items used by <25% of the children, items with considerable differences by age (23–25 months) and those with a positive predictive value below 50% without significant reduction of predictive quality with regard to a preliminary definition of SLD.

**Table 1:** Random and total sample characteristics.

	**A**	**B**	**C**	**Difference between B and C^c^**
	**Population^b^**	**Full sample**	**Analysis sample**	
	**(*n* = 13,297)**	**(*n* = 2,044)**	**(*n* = 621)**	
Child age M (SD)		23.92 (0.993)	23.92 (0.972)	*d* = 0.00, *p* > 0.05
Child sex (male)^d^ %	51.1%	50.9%	51.8%	ϕ = 0.01, *p* > 0.05
Premature birth^e^ %	8.7%	8.6%	8.7%	ϕ =0.00, *p* > 0.05
				
***Highest parental education**^a^^f^ **%***				
Compulsory education (or below)	7%	2.2%	1.4%	Cramer's V = 0.05, *p* > 0.05
Vocational education	47%	47.7%	47.0%	
University entrance	21%	23.5%	25.8%	
University	25%	26.6%	25.8%	
				
***Parent reports***				
Expressive vocabulary M (SD)		132.75 (65.617)	134.94 (64.789)	*d* = −0.04, *p* > 0.05
No two-word combinations %		6.9%	5.6%	ϕ = −0.02, *p* > 0.05
Parental concerns (yes) %		13.2%	12.4%	ϕ = −0.02, *p* > 0.05
				
Pediatric assessment (Age 2) M (SD)		6.91 (1.747)	6.86 (1.796)	*d* = 0.05, *p* > 0.05
				
Significant language deficit (−1.5 SD at age 3)^g^ %		9.1%	11.3%	ϕ = 0.05, *p* < 0.05

a The highest education of the two parents was used.

b Population values for child sex and premature birth are taken from (43) and directly refer to Upper Austrian birth cohort of 2007. Due to the lack of population data for parental education directly referring to the birth cohort of 2007, we use parental education values from the parent population of Upper Austrian 4. graders of 2018 as proxies (44). Notably, these values also include Non-German speaking parents. Detailed data by language use for Upper Austria are not available.

c p-values for categorical variables refer to χ^2^-tests (effect size ϕ or Cramer's V). p-values for continuous variables refer to t-tests (effect size d).

d Difference between A and B: ϕ = 0.00, p > 0.05;

e Difference between A and B: ϕ = 0.01, p > 0.05;

f Difference between A and B: Cramer's = 0.20, p < 0.001.

In total, 46 children were excluded due to incomplete data on the parent reports (*n* = 4) and pediatrician assessment (*n* = 40). Thus, the final sample used for the analyses in this study comprised 621 children (mean age = 23.92 months, SD = 0.972; 51.8 % were boys, 48.2% were girls). Descriptive statistics for the study variables are reported in Table 1. About 11.5% of the children were regarded as having significant language deficits (SLD) at the age of 3 years based on the reference test. Notably, the final sample (*n* = 621) used in this study did not substantially deviate from the full sample (*n* = 2,044; see Table 1 for details).

### Measures

#### Screening measures

The final screening measures (SPES-2, Sprachentwicklungsscreening-2; language screening for 2-year-old children) used in the validation study included a parent-reported questionnaire with demographic information on the child (age, sex, age at completion of questionnaire, birth order, child's best language) and the family (maternal and paternal education, primary family language). Other information included parental estimation of the child's language development, parental concerns about language development, the 37 items on expressive vocabulary, and a question on two-word-combination use.

Since word comprehension was found to contribute to the prediction of SLD in the pilot study, a word comprehension subtest of a standardized German language test [SETK-2; (46)] was selected for the direct pediatric assessment. The pediatrician directs single words (9 items; Cronbach's Alpha = 0.69) referring to household items, food, animals and body parts to the child, and the child is asked to identify the corresponding picture from four options.

#### Reference test for significant language deficits at the age of 3 years

In the absence of a well-defined standard for diagnosing language disorder, we used the SPES-3 language screening measure (47) as reference test for SLD at the age of 3 years. The SPES-3 language screening—consisting of parent reports on expressive vocabulary and expressive grammar—is administered during regular well-baby check-ups at the age of 3 years. The SPES-3 screening tool has an excellent diagnostic accuracy (AUC = 0.946) in predicting language disorder (assessed by two experienced clinical linguists blinded to the SPES-3 result). For this study, a cutoff of 1.5 SD below the mean of the total SPES-3 screening score was used [for details see (47)].

#### Feasibility measures

Feasibility was measured by use of a questionnaire completed by the participating pediatricians. The questionnaire was designed following the guidelines by Bowen et al. (48). It included three components on feasibility, with all questions rated on a 4-point Likert scale (very good, good, difficult, and very difficult). Practicality concerns the extent to which administration of the screening was considered possible within regular pediatric primary care. In addition, pediatricians were asked to report on the ease of administration of the direct assessment and parental difficulties in completing the parent-reported questionnaire. Additionally, pediatricians ranked five pre-specified factors that might challenge the administration of the screening tool. Acceptability refers to the reactions to the screening tool by the children, parents, and pediatricians, including the proportion of direct screening tests that could be completed by the pediatricians. The pediatricians were asked to assess parental acceptance of the inclusion of language screening in the medical check-up of children at the age of 2 years, and assess the language screening tool's usefulness. Finally, sustainability was assessed by asking the pediatricians whether they intended to continue language screening after the research project ended.

### Analytic strategy

A central aim of this study was to develop a two-stage screening procedure made up of an assessment based on parent report (stage 1) followed by pediatric direct assessment (stage 2) only for cases with atypical results in the first screening stage. To achieve this aim, we used in a first step logistic regression to predict SLD at the age of 3 years using the set of parent-reported language-related variables (expressive vocabulary list, parental concerns, two-word combinations) and child and parent-related sociodemographic variables (child age, child sex, parental education) as possible predictors.

Significant predictors were identified using a backward variable selection algorithm (Likelihood Ratio).

Subsequently, a stage 1 screening score was computed as the probability of having SLD based on the logistic regression results.

Screening failures at stage 1 that would undergo direct pediatric assessment at stage 2 were determined by selecting a cutoff point with a quite high sensitivity of 0.90. Thus, the group of stage 1 screening failures covers 90% of all children with SLD. Commonly, a sensitivity of 0.80 is regarded as acceptable for developmental screenings (49). In their meta-analysis of predictive language screening tools at the pre-school age, Sim et al. (28) reported a mean sensitivity of 0.66 (range, 0.52–0.80).

In order to improve the SLD-prediction for children who failed screening stage 1, again logistic regression was used but—in addition to the significant predictors of stage 1—the word comprehension subtest of the SETK-2 was entered as additional predictor into the model. Thus, we evaluated whether the direct pediatric assessment significantly contributes to the prediction of SLD at the age of 3 years for children who failed screening at stage 1. In the case of an incremental contribution of the pediatric assessment to the SLD-prediction, a new (stage 2) screening score (probability of having SLD) for children failing the stage 1 screening was computed.

Subsequently, the diagnostic accuracy of the 2-stage screening procedure (i.e., stage 1 score for screening passes at stage 1 and stage 2 score for screening fails at stage 1) was evaluated and compared with simple stage 1 screening by applying ROC (receiver operating characteristics) analyses and AUC difference tests for paired ROC curves. AUCs ≥0.9 are regarded as excellent, AUCs ≥0.8 and <0.9 as good, AUCs ≥0.7 and <0.8 as fair, and tests with AUCs < 0.7 as poor (50).

Finally, to provide information about graduations of risk for SLD, cutoffs for the high-risk and moderate-risk groups were evaluated by estimating the following diagnostic efficiency statistics: sensitivity (Se), specificity (Sp), positive predictive values (PPV), negative predictive values (NPV), and diagnostic likelihood ratios for positive and negative screening results (DLR+ and DLR-). DLRs are alternative measures of diagnostic accuracy and display the multiplicative change in the pre-screening odds of having SLD given a positive (DLR+) or negative screening result (DLR–). DLR+ values ≥10 and DLR– ≤ 0.1 indicate large changes in pre-screening odds. DLR+ ≤ 10 and >5 and DLR– >0.1 and ≤ 0.2 indicate moderate changes; DLR+ ≤ 5 and >2 and DLR– >0.2 and ≤ 0.5 indicate small changes. DLR+ <2 and DLR– >0.5 are rarely important (51). To further evaluate any advantages of the 2-stage screening procedure, cutoffs were estimated for the simple stage 1 screening and the 2-stage screening, and subsequently compared in terms of their diagnostic efficiency. The cutoff for the high-risk group was determined by fixing PPV at 0.80; that is, children who failed to meet this cutoff had a probability of 80% of having SLD about 1 year after the screening. The PPV of 0.80 was selected for pragmatic reasons taking the limited availability of follow-up diagnostic services and the high probability of need for early intervention into account. For identifying a moderate-risk group, various cutoffs corresponding to sensitivities of 0.75, 0.80, and 0.85 were selected. Notably, as a sensitivity of 0.90 was chosen at stage 1, cutoffs with high sensitivity values (near at least 0.90) in the 2-stage screening process would, by design, hardly differ from stage 1 screening. Figure 1 summarizes the conceptual design of the 2-stage screening process described above.

Logistic regressions and descriptive analyses were performed using SPSS 27 (28, 52). Furthermore, the pROC package in R (53) was used to perform ROC analysis and tests for paired ROC curves. Additionally, the R-OptimalCutpoints package (54) was used to estimate cutoffs and diagnostic efficiency statistics.

## Results

Before presenting the results of the two-stage screening, we compared the diagnostic accuracy of the 37 item word list with the initial 260 word list with regard to the SLD measure used in this study. Diagnostic accuracy (AUC) was 0.866 [DeLong 95%CI (0.827–0.905)] for the 260 item list and 0.857 [DeLong 95%CI (0.814–0.900)] for the 47 item list. As indicated by a bootstrapped test for comparing paired ROC curves, the difference was not significant (ΔAUC = 0.009, D = 0.721, *p* = 0.471).

### Stage 1 screening

The results of the logistic regression to identify significant parent-reported predictors of SLD at the age of 3 years are shown in Table 2. In the first results column, the estimates for the full model are reported, i.e., the model in which all variables were entered simultaneously as predictors. The second column (Final Model) shows the estimates after excluding non-significant predictors (logistic regression-based backward elimination). Notably, as in the full model, only parental concerns [b = 1.231, *p* < 0.001, odds ratio (OR) = 3.424 95% CI (1.713, 6.843)], no two-word combinations [b = 1.268, *p* < 0.01, OR = 3.554 95% CI (1.409, 8.963)], and expressive vocabulary [b = −0.088, *p* < 0.001, OR = 0.916 95% CI (0.888, 0.944)] significantly predicted SLD. Based on these results, a stage 1 screening score (i.e. probability of SLD) was calculated.

**Table 2:** Logistic regression predicting LD at age 3 – Stage 1.

	**Full model**			**Final model**		
	**b**	**SE**	**OR (95%CI)**	**b**	**SE**	**OR (95% CI)**
Parental highest education (reference = university)^a^			
*Compulsory education*	1.782*	0.895	5.940 (1.029, 34.304)			
*Vocational education*	0.325	0.425	1.384 (0.602, 3.182)			
*University entrance*	0.164	0.480	1.178 (0.460, 3.018)			
Child gender (1 = male)	0.016	0.317	1.016 (0.546, 1.892)			
Child age	0.211	0.160	1.235 (0.903, 1.690)			
Parental concerns	1.176***	0.366	3.243 (1.583, 6.645)	1.231***	0.353	3.424 (1.713, 6.843)
No two-word utterances	1.268**	0.477	3.552 (1.394, 9.055)	1.268**	0.472	3,554 (1,409, 8.963)
Expressive vocabulary	−0.091***	0.016	0.913 (0.884,0.943)	−0.088***	0.016	0.916 (0.888,0.944)
Intercept	−6.485	3.834		−1.217	0.777	
R^2^ Nagelkerke	0.365			0.351		

a Overall p-value based on Wald test > 0.05.

The *, **, and *** symbols indicate the value of p < 0.05, p < 0.01, and p < 0.001 respectively.

To identify children that should also undergo screening stage 2, a cutoff was determined by fixing sensitivity at 0.90. The cutoff of 0.05 on a probability scale met this requirement and yielded the following accuracy statistics: Se = 0.900 [95% CI (0.805, 0.959)], Sp = 0.708 [95% CI (0.668, 0.745)], PPV = 0.281 [95% CI (0.668, 0.745)], NPV = 0.982 [95% CI (0.962, 0.985)], DLR+ = 3.080 [95% CI (2.646, 3.584)], and DLR– = 0.141 [95% CI (0.070, 0.286)]. Notably, as expected, the high sensitivity of 0.90 was at the expense of a quite low PPV. Given this cutoff, 224 (36%) children failed the stage 1 of the screening process and thus, are considered in stage 2 screening.

### Stage 2 screening

To evaluate whether the pediatric assessment incrementally contributes to the prediction of SLD in children failing stage 1 (*N* = 224), a logistic regression model including all selected predictors of stage 1 and additionally the pediatric scale of word comprehension, was used. The results are reported in Table 3.

**Table 3:** Logistic regression predicting LD at age 3 – Stage 2.

	**b**	**SE**	**OR (95% CI)**
Parental concerns	0.721*	0.352	2.056 (1.031, 4.100)
No two-word utterances	1.159**	0.448	3.185 (1.323, 7.667)
Expressive vocabulary	–0.038*	0.018	0.962 (0.928, 0.997)
Word comprehension	–0.284***	0.084	0.753 (0.639, 0.887)
Intercept	–0.069	0.882	
R^2^ Nagelkerke	0.282		

The *, **, and *** symbols indicate the value of p < 0.05, p < 0.01, and p < 0.001 respectively.

All three parent-reported stage 1 predictors and also the pediatric assessed word comprehension significantly contributed to the predicting of SLD. Notably, as indicated by the standardized coefficients, word comprehension had the greatest predictive power (stand. *b* = −0.590, vs. stand. *b* = 0.422 for two-word combinations, stand. *b* = 0.343 for parental concerns and stand. *b* = −0.365 for expressive vocabulary). Like in stage 1, the predicted probability of having SLD was calculated based on the regression results. Subsequently, a *total two-stage screening score* was computed. This score equals the stage 1 score for children who passed stage 1. For children who failed the screening at stage 1, the predicted probability of stage 2 was used.

### Comparing diagnostic accuracies between the total two-stage screening and one-stage screening tool

ROC analyses yielded an AUC of 0.875 [DeLong 95% CI (0.833–0.917)] for the one-stage screening (i.e., screening score of stage 1), and a slightly better AUC of 0.885 [DeLong 95% CI (0.843–0.926)] for the total two-stage screening. A bootstrapped test for comparing paired ROC curves shows that the AUC difference (ΔAUC = 0.01) was marginally significant (D = 1.718*, p* = 0.086).

### Cutoff estimation

Finally, various cutoffs were estimated, and the respective diagnostic efficiencies for the total two-stage screening tool and one-stage screening tool were evaluated (see Table 4). To identify a high-risk group that is characterized by a high probability of having a SLD, the cutoffs were estimated by fixing the PPV at 0.8. For the one-stage and two-stage screening tools, a cutoff of 0.758 (2.4% screening failures) and 0.594 (4% screening fails), respectively, resulted in a PPV of 0.80. Given the same PPV, the sensitivity of the total two-stage screening [0.286, 95% CI (0.092, 0.280) ]tool was significantly higher than that of the one-stage screening tool [0.171, 95% CI (0.183, 0.406)]; McNemar test χ^2^ (1) = 6.125, *p* = 0.008. Thus, the total two-stage screening tool identifies a larger proportion of children with SLD than the one-stage screening tool.

**Table 4:** Diagnostic accuracy statistics for different cutoffs (95% CIs).

	**Cutoff**	**%Fails**	**Sens**	**Spec**	**PPV**	**NPV**	**DLR+**	**DLR-**
**High Risk**								
Stage 1 screening	0.758	2.4%	0.171	0.995	0.800	0.904	31.486	0.833
			(0.092, 0.280)	(0.984, 0.999)	(0.577, 0.883)	(0.822, 0.979)	(9.107, 108.853)	(0.749, 0.927)
Total 2-stage screening	0.594	4.0%	0.286	0.991	0.800	0.916	31.486	0.721
			(0.184, 0.406)	(0.979, 0.997)	(0.630, 0.872)	(0.860, 0.971)	(12.202, 81.242)	(0.621, 0.836)
**Moderate Risk & High Risk (Se = 0.75)**				
								
Stage 1 screening	0.089	24.6%	0.729	0.815	0.333	0.959	3.936	0.333
			(0.609, 0.828)	(0.780, 0.846)	(0.287, 0.473)	(0.932, 0.967)	(3.139, 4.934)	(0.226, 0.490)
Total 2-stage screening	0.169	20.5%	0.743	0.864	0.409	0.964	5.458	0.298
			(0.624, 0.840)	(0.832, 0.891)	(0.352, 0.557)	(0.938, 0.972)	(4.244, 7.018)	(0.200, 0.444)
**Moderate Risk & High Risk (Se = 0.80)**				
Stage 1	0.076	28.0%	0.788	0.797	0.329	0.967	3.865	0.269
			(0.671, 0.875)	(0.761, 0.830)	(0.285, 0.483)	(0.942, 0.973)	(3.147, 4.748)	(0.171, 0.422)
Total 2-stage screening	0.128	26.7%	0.800	0.786	0.322	0.969	3.736	0.255
			(0.687, 0.886)	(0.749, 0.819)	(0.279, 0.480)	(0.944, 0.975)	(3.064, 4.555)	(0.159, 0.407)
**Moderate Risk & High Risk (Se = 0.85)**				
Stage 1	0.059	30.6%	0.843	0.762	0.311	0.974	3.545	0.206
			(0.736, 0.919)	(0.724, 0.797)	(0.270, 0.487)	(0.952, 0.979)	(2.960, 4.246)	(0.120, 0.355)
Total 2-stage screening	0.122	29.1%	0.843	0.779	0.326	0.975	3.807	0.202
			(0.736, 0.919)	(0.742, 0.813)	(0.283, 0.505)	(0.953, 0.980)	(3.159, 4.587)	(0.117, 0.348)

Subsequently, an additional moderate-risk group, which together with the high-risk group should cover 75% of the children with SLD (i.e., sensitivity = 0.75), was identified. Cutoff values that achieved a sensitivity closest to 0.75 were 0.089 (24.6% screening failures; Se = 0.729, 95%CI (0.609, 0.828) for the one-stage screening tool and 0.169 (20.5% screening failures; Se = 0.743, 95% CI (0.624, 0.840) for the two-stage screening tool. Notably, the total two-stage screening tool [Sp = 0.864, 95% CI (0.832, 0.891)] was more specific than the one-stage screening (Sp = 0.815, 95% CI (0.780, 0.846); McNemar Test χ^2^ (1) = 11.860, *p* < 0.001.

Further cutoffs for the moderate risk group associated with higher sensitivity were also evaluated (see Table 4). Overall, the differences between the one-stage and two-stage screening tools decreased for higher sensitivity values.

**Figure 2:**
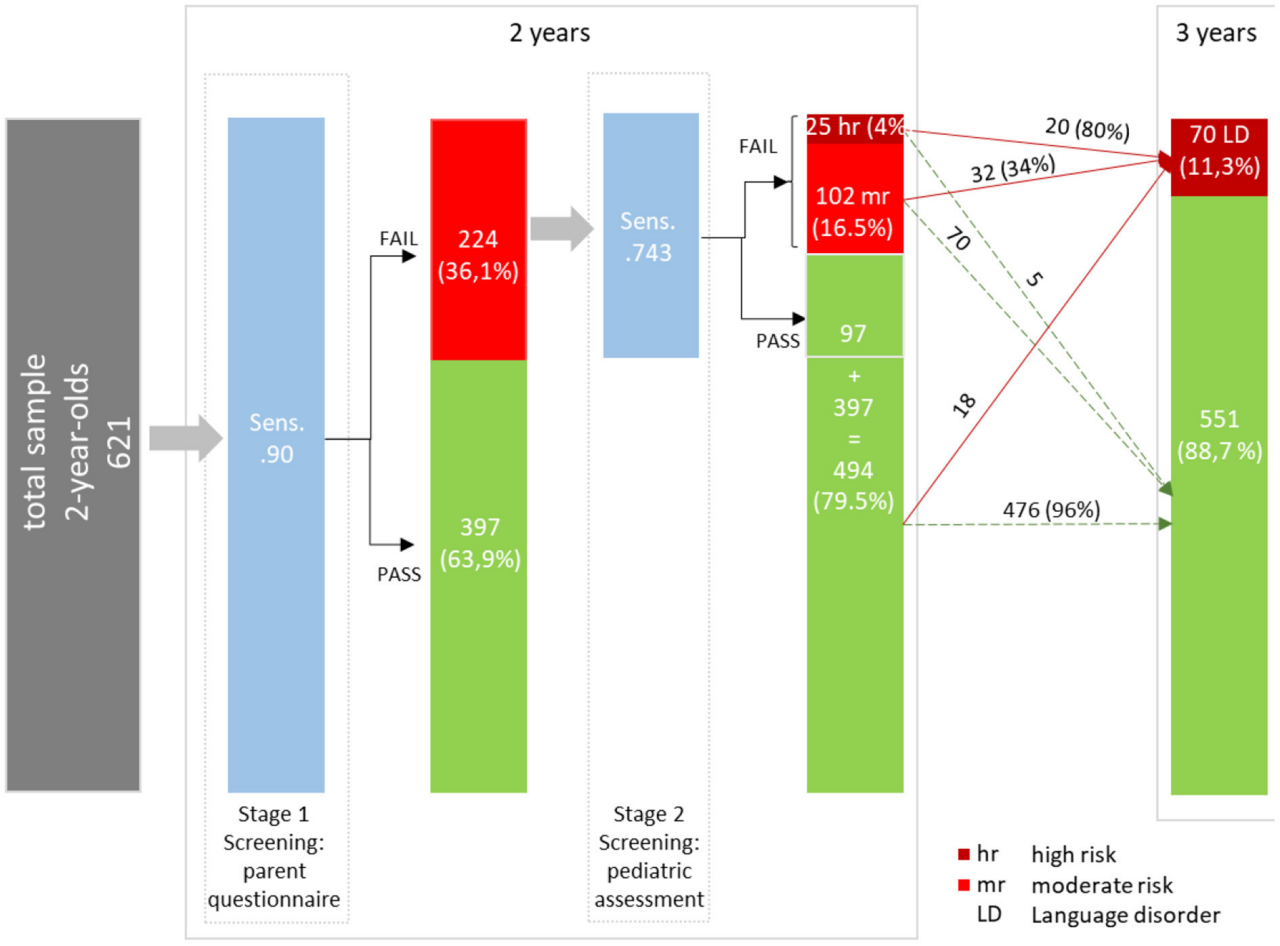
Overview of results of the two-stage screening tool.

Figure 2 summarizes the results of the proposed two-stage screening tool. At stage 1, the high sensitivity of 0.90 was accompanied by quite a high rate of screening failures (36.1%) and consequently with a rather large share of false positives (PPV = 0.281). After stage 2, a small group of 4% is classified as high-risk for SLD. Twenty out of these 25 children are classified as having SLD at the age of 3 years (PPV = 0.80). Notably, their pre-screening odds (before stage 1 screening) of having an SLD increases by about 31 times (DLR+ = 31.486), given a screening failure (high risk) at stage 2. However, the high-risk group only accounts for 20 out of 70 children with SLD at the age of 3 years. Thus, the high-risk cutoff is associated with a low sensitivity (28.6). The moderate risk group (16.5%) covers further 32 children with SLD at the age of 3 years (sensitivity = 0.457). The change in the pre-screening odds of having SLD for children in the moderate-risk group (DLR+ = 3.360) is minimal. The true positives from the high-risk and moderate-risk groups sum up to 52 (Sensitivity = 0.743) children. Finally, 476 out of 494 children with no SLD at the age of 3 years are correctly identified by the two-stage screening tool (specificity = 0.864).

### Supplementary analyses

Finally, to validate the differentiation between a low, moderate and high-risk group, we performed a set of supplementary analyses. In detail, we compared the means of the two reference test scales (expressive vocabulary and expressive grammar; see measures section), between the three groups.

We found that the high-risk group [expressive vocabulary: *M* = −1.73, standard deviation (SD) = 0.945; expressive grammar: *M* = −1.99, SD = 1.020] scored about one SD lower than the moderate-risk group (expressive vocabulary: *M* = −0.791, SD = 1.030; expressive grammar: *M* = −1.016, SD = 1.193) and about two SDs lower than the remaining low-risk group (expressive vocabulary: *M* = 0.255, SD = 0.826; expressive grammar: *M* = 0.260, SD = 0.804). Thus, all groups substantially differed in their means [Expressive vocabulary: *F*_(2, 52.671)_ = 88.344, *p* < 0.001; η^2^ = 0.262].

### Feasibility

Twenty-three out of the 30 pediatricians (77%) who participated in the study returned completed questionnaires on feasibility of SPES-2 language screening.

#### Practicality

Ease of administration of the word comprehension screening was rated as “very good” and “good” by 52 and 48%, respectively, of the pediatricians. Thus, none of the pediatricians rated ease of administration as “difficult.” Ease of integrating the screening within the time constraints of regular pediatric care was mostly rated as “good” (65%), as “very good” in 26%, and only one pediatrician regarded it as “difficult.” Among the five pre-specified factors that might possibly challenge the administration of language screening within primary pediatric care, lack of or insufficient follow-up was ranked highest (40%). Insufficient training of pediatricians and lack of or insufficient funding were equally regarded as most challenging by 20%, followed by limited meaningfulness of language screening (13%) and time constraints (4%).

#### Acceptability

Parental acceptance of language screening included within the regular well-baby check-up at the age of 2 years was rated as either “very good” (57%) or “good” (43%) by the pediatricians. The acceptance of the questionnaire by parents was rated as “good” by the majority (52%), as “very good” by 35%, and as “difficult” by 13% of pediatricians. Most (77%) pediatricians rated the meaningfulness of language screening within the regular preventive check-ups as “very good,” 14% as” good,” and only 9% expressed concerns.

#### Sustainability

Most of the pediatricians (83%) indicated that they would continue the SPES-2 language screening beyond the research project, whereas 17% would not. Notably, no additional funding for the language screening study was provided by the public health system.

## Discussion

This study evaluated the screening accuracy and feasibility of a newly developed language screening tool (SPES-2) for 2-year-old children in a large population sample within regular well-baby check-ups in primary pediatric care in Upper Austria. The two-stage screening tool included parent-reported information on the child's expressive vocabulary, production of two-word-combinations, and parental concerns about language development during stage 1. During stage 2, results of the pediatrician's assessment of word comprehension demonstrated good diagnostic accuracy ((AUC = 0.885) for the prediction of SLD about 1 year after the screening (for children at the age of 3 years).

The two-stage screening tool identified a significantly larger proportion of children ending up with SLD at the age of 3 years than the first screening stage (parent reports) only. Defining a high-risk group by a probability of 80% of ending up with SLD at age of 3 years, 4% of the study sample were identified, representing 28.6% of the children with SLD at the age of 3 years. Another 16.5% of the total sample were identified as a group with moderate risk for SLD at the age of 3 years (risk increased three-fold). The moderate-risk group comprised 45.7% of the children with SLD 1 year later. Thus, both risk groups (high and moderate: 20.5% of all children) included about 75% of all children diagnosed with SLD at the age of 3 years (sensitivity: 0.743, and specificity: 0.864). The classification of screening results by degree of risk (low-moderate-high) was also supported by significant differences (of about 1 standard deviation) among the three groups.

Our findings of high predictive validity of the screening tool after 1 year confirm those of the few language screening tools in 2-year-old children that predict later language status. The Dutch well-child language screening protocol for 2-year-old children (29), which is based on direct-child assessment of word comprehension and parent reports (word combinations and playing behavior), yielded a slightly higher sensitivity (0.82) but lower specificity (0.74) to predict language problems 1 year later. However, about half of the validation sample were screening failures, and referral bias artificially increases sensitivity. Predictive sensitivity and specificity reported for the German MCDI-based ELFRA (34) (parent questionnaire for 2-year-old children) were 0.61 and 0.94, respectively (55). However, generalizability to the total population is again limited due to a high overrepresentation of screening failures (60%) in the study sample. For the Language Development Survey (56), predictive validity was similar to that found by the ELFRA study with sensitivity of 0.67 and specificity of 0.96. However, only 15.6% of the LDS sample were included in the follow-up assessment that was performed at an average of only 23 days after the screening. Given the high variability of trajectories of early language development, a short time lag between screening and follow-up certainly contributes to its predictive validity. The screening battery approach by Stott et al. (57) applied the General Language Screen and Developmental Profile II at the age of 36 months to predict speech and language disorders at 45 months of age. Both, sensitivity (0.67) and specificity (0.68) were significantly lower than those achieved by the SPES2 measure.

Thus far, predictive validity of parental reports of expressive vocabulary, two-word-combinations, parental concerns, and language comprehension aligns with the international evidence (55, 56).

The graduation of risk (high vs. moderate) of screening failures allows for tailored follow-up and intervention, avoiding a high rate of over-referrals related to the lower specificity of SPES-2 than MCDI and LDS. Significant differences of results of standardized language tests between the group with no and moderate risk (about−1SD) and between the moderate and high-risk groups (about−1SD) confirm the validity of the three risk levels requiring different follow-up procedures. For the high-risk group, follow-up assessments of language, hearing, cognitive, and psycho-social development should be considered, because the combination of expressive and receptive language difficulties can be indicative of more comprehensive or pervasive developmental disorders, such as general developmental delay or autism spectrum disorders. For the group with moderate but still significantly increased risk of SLD, preventive parent counseling or parent training (for example, promoting a responsive interaction style with their children and facilitative language techniques within everyday family routines or dialogic book reading) seems to be indicated. Due to the instability of early language development between the ages of 2 and 3 years, continuous language surveillance (for example, language screening at the age of 3 years) should be recommended to *all* parents including those of children in the low-risk group. The severity and type of language disorders and the prevalence of other neurodevelopmental disorders in the subgroups of children with high, moderate, or low risk still need to be determined to further substantiate the risk levels resulting from the language screening.

The high feasibility of the new screening tool within primary pediatric care was not anticipated. The two-stage procedure, including an initial collection of risk indicators from parents followed by a very short and easy-to-score direct assessment of word comprehension in only about one-third of the children, undoubtedly contributes to the high practicality. For the majority of pediatricians, the implementation of language screenings within regular well-baby check-ups appeared appropriate. However, factors possibly challenging the implementation of language screenings, particularly the provision of follow-up diagnostic assessment and parental guidance, specific training of pediatricians, and funding of the screening procedures, need to be considered in the planning of a population-based realization.

Our study had some major strengths. First, the screening was designed to predict language disorders 1 year after its implementation. Second, the non-selected total population sample of 2-year-old children were assessed within regular pediatric well-baby check-ups. Third, all the children, regardless of their screening results, were followed up by use of validated language measures at the age of 3 years, which highly restricting potential bias in our study. Fourth, the inclusion of feasibility in the evaluation of the screening is, as an essential contribution to the field (19), with a demand of information about practicality and acceptability. However, the lack of multidimensional standardized follow-up diagnostic assessments on a random selection of children with high, moderate, and low risk of SLD can be considered a limitation of this study. Another limitation was the voluntary participation of pediatricians that might have positively influenced feasibility. Exclusion of bilingual children was only based on parent information excluding children with a non-German language peferrably used in their families Therefore, children growing up with another language in addition to the primary German family language were included in the study samples. The use of a second language might have an influence on their German language development that is no taken into account in the current study.

## Conclusion

Our findings on predictive sensitivity and specificity of the language screening tool, SPES-2, demonstrate its validity for the early identification of SLD in 2-year-old children that persist to the age of 3 years. The two-stage procedure of parental report followed by direct pediatric assessment only in those who failed the first screening stage makes the screening time-efficient. The grading of risk levels derived from screening results supports tailored follow-up and requires further clinical validation.

## Data availability statement

The raw data supporting the conclusions of this article will be made available by the authors at request, without undue reservation.

## Ethics statement

The studies involving human participants were reviewed and approved by Ethikkommission Konventhospital Barmherzige Brüder Linz. Written informed consent to participate in this study was provided by the participants' legal guardian/next of kin.

## Author contributions

DH and JF: conceptualization and investigation. DH, CW, and JF: methodology, original draft preparation, review, and editing. CW: formal analysis. DH: data curation and project administration. All authors contributed to the article and approved the submitted version.

## Funding

This study was financially supported by the Department of Health and Social Affairs of the Upper Austrian government. Article Processing Charge is funded by the Johannes Kepler University Open Access Publishing Fund.

## Conflict of interest

The authors declare that the research was conducted in the absence of any commercial or financial relationships that could be construed as a potential conflict of interest.

## Publisher's note

All claims expressed in this article are solely those of the authors and do not necessarily represent those of their affiliated organizations, or those of the publisher, the editors and the reviewers. Any product that may be evaluated in this article, or claim that may be made by its manufacturer, is not guaranteed or endorsed by the publisher.
