# Supplementary material for: Validity and feasibility of a predictive language screening tool in 2-year-old children in primary pediatric care
Source: Front Pediatr. 2022 Sep 6;10:865457. doi: 10.3389/fped.2022.865457 (PMC9485630; doi:10.3389/fped.2022.865457)
Supplement: Supplementary file 1 [file Data_Sheet_1.PDF]

HOW DOES YOUR CHILD TALK?

Dear parents!

Language development is an important foundation for your child's overall development, especially for later academic learning, development of relationships and mental health. In this context, the first years of life are of particular importance. To assess the level of language development of a 2-year-old child, observations by parents are crucial. We therefore ask you to fill out this form completely.

child's name ___________________________ surname ____________________________ name

child's sex male ☐ female ☐

date of birth __________________________ **today's date** __________________________

day / month / year day / month / year

Family

What is the child's birth order? ____ (e.g. 1., 2. 3. …)

Parents' education (multiple answers permitted)

Mother

☐ no graduation/special school qualification
☐ basic school qualification/compulsory education
☐ apprenticeship
☐ college
☐ high school qualification
☐ university

Father

☐ no graduation/special school qualification
☐ basic school qualification/compulsory educ.
☐ apprenticeship
☐ college
☐ high school qualification
☐ university

Language

What is the preferred language in the family?

☐ German

☐ other: ____

If other: Which language does your child speak best? ______________________________

Assessment of Language Development

At what age could your child speak 5 words?

_________ ; __________ □ speaks less than 5 words currently

Year(s) months

How would you assess the development of your child's native language compared to children of the same age?

□ normal □ slightly delayed □ strongly delayed

Have you been concerned about your child's language development?

□ no □ a little □ yes, a lot

Wordlist

Children can usually already understand many more words than they speak. However, this part of the questionnaire is about which words your child already **SPEAKS**. Please mark the words that you have already heard spontaneously from your child by ticking the circle.

- *If your child uses a slightly different pronunciation (e.g. "tow" instead of "cow"; "tator" instead of "tractor"), please mark the respective word anyway.*
- *If your child uses a substitution instead of the target word (e.g., "meow" instead of "cat"; "yum yum" instead of "eat") or doesn't use the word at all yet, please just leave the circle blank.*

dog	○	rabbit	○	pants	○	bear	○
car	○	shoes	○	mouth	○	duck	○
cat	○	house	○	egg	○	stone	○
baby	○	doll	○	sheep	○	bread	○
cow	○	ball	○	tractor	○	juice	○
mom(my)	○	apple	○	hand	○	horse	○
bird	○	book	○	ear	○	milk	○
water	○	tree	○	bed	○	**TOTAL**	
eat	○	eyes	○	flower	○		
nose	○	table	○	glasses	○		

Institut für Sinnes- und Sprachneurologie
Konventhospital Barmherzige Brüder
Seilerstätte 2, A-4021 Linz; nla@bblinz.at

BARMHERZIGE BRÜDER
KONVENTHOSPITAL LINZ

WORD COMBINATIONS

Has your child already started to combine words?

e.g.. „Peter play“, „drink juice“

□ yes

□ not yet

Can you give two examples of the longest sentences your child currently says?

1. ____

2. ____

CONSENT FORM

I agree that my child's data may be used anonymously for research purposes to improve early detection of language problems.

□ yes

□ no

____

parent’s name & surname

________________________ ________________________

place, date parent's signature

THANK YOU VERY MUCH FOR YOUR COOPERATION!

THE NEXT PAGE IS FILLED IN BY THE PEDIATRICIAN

Institut für Sinnes- und Sprachneurologie
Konventhospital Barmherzige Brüder
Seilerstätte 2, A-4021 Linz; nla@bblinz.at

BARMHERZIGE BRÜDER
KONVENTHOSPITAL LINZ

ASSESSMENT OF COGNITIVE DEVELOPMENT

□ normal □ slightly delayed □ strongly delayed (AER)

WORD COMPREHENSION (SET-K 2)

Instruction

Here I have got some pictures for you. Let's do it like this: I tell you something and you always show me the right picture. Only one picture is correct at a time. Let's try it out now. Show me: rabbit.

picture 1 *(Exercise)*	**rabbit**		
picture 2	**cheese**	R	F
picture 3	**scissors**	R	F
picture 4	**pen**	R	F
picture 5	**mouth**	R	F
picture 1	**cow**	R	F
picture 2	**carrot**	R	F
picture 3	**knife**	R	F
picture 4	**cupboard**	R	F
picture 5	**nose**	R	F
	**TOTAL**		

Institut für Sinnes- und Sprachneurologie
Konventhospital Barmherzige Brüder
Seilerstätte 2, A-4021 Linz; nla@bblinz.at

BARMHERZIGE BRÜDER
KONVENTHOSPITAL LINZ
